# Supplementary material for: Bone and joint infections due to melioidosis; diagnostic and management strategies to optimise outcomes
Source: PLoS Negl Trop Dis. 2024 Jul 17;18(7):e0012317. doi: 10.1371/journal.pntd.0012317 (PMC11253972; doi:10.1371/journal.pntd.0012317)
Supplement: S1 Table — (DOCX) [file pntd.0012317.s001.docx]

**Supplementary table 1.** Demographic and clinical characteristics, management, and clinical course of the 39 individuals with bone and joint infections due to *Burkholderia pseudomallei*

| Study number | Age/sex | FNA | Remote residence | Other risk factors | OM, SA or both | Site | Initial Presentation | Other organs involved | Bacteremia | Number of operations | IV agent(s) | PO agent(s) | Complications |
| --- | --- | --- | --- | --- | --- | --- | --- | --- | --- | --- | --- | --- | --- |
| 6 | 39F | Yes | Yes | DM | SA | Ankle joint | 1° BJI | Liver, spleen, pancreas, kidney, lung | Yes | 1 | Ceftazidime | TMP-SMX | - |
| 13 | 61F ^a^ | Yes | Yes | DM | SA | Knee joint | 1° BJI | - | No | 2 | Ceftazidime | TMP-SMX | - |
| 94 | 34M | Yes | Yes | DM | Both (adjacent) | Ankle joint, talus | 1° BJI | - | Yes | 2 | Meropenem | TMP-SMX | - |
| 127 | 32F | Yes | Yes | DM | Both (adjacent) | Ankle joint, tibia | 1° BJI | - | No | 4 | Meropenem | TMP-SMX | Sinus |
| 141 | 57M | No | No | Nil | OM | Hallux | 1° BJI | - | No | 0 | Ceftazidime | TMP-SMX | - |
| 199 | 55M | Yes | Yes | DM, EtOH | OM | Femur | 1° BJI | - | Yes | 2 | Meropenem + ceftazidime | TMP-SMX | Readmission for culture- confirmed relapse |
| 256 | 29M | Yes | Yes | DM, EtOH, CLD | Both (adjacent) | Knee joint, patella | 1° BJI | Lung | No | 1 | Meropenem | TMP-SMX | Septic shock |
| 258 | 34M | Yes | Yes | DM | OM | Tibia | 1° BJI | Lung, liver | Yes | 0 | Ceftazidime | TMP-SMX | - |
| 305 | 42M | No | Yes | EtOH | Both (adjacent) | Knee joint, tibia | 1° BJI | Lung | Yes | 3 | Ceftazidime | TMP-SMX | - |
| 405 | 43M | Yes | Yes | DM | Both (non-adjacent) | Ankle joint, wrist joint, tibia, metatarsal, ulna | 1° BJI | Lung, SSTI | Yes | 8 | Meropenem | TMP-SMX | - |
| 26 | 31F | Yes | No | DM, EtOH, immunosuppression | Both (non-adjacent) | Knee joint, glenohumeral joint, humerus | 2° BJI | Lung, genitourinary, liver, spleen, SSTI | Yes | 7 | Meropenem + ceftazidime | TMP-SMX | Arthroscopic converted to open. Readmission for culture-confirmed recrudescence |
| 46 | 45M | Yes | Yes | DM, EtOH | OM | Calcaneus, talus | 2° BJI | Prostate, spleen | Yes | 0 | Ceftazidime | TMP-SMX | Pathological fracture |
| 69 | 53M | Yes | Yes | DM, EtOH | Both (non-adjacent) | Ankle joint, calcaneus, femur | 2° BJI | Lung | Yes | 1 | Meropenem + ceftazidime | TMP-SMX | Readmission for clinically suspected recrudescence. ^b^ |
| 76 | 56M | Yes | Yes | DM, EtOH | Both (adjacent) | Subtalar joint, tibia, talus, calcaneus | 2° BJI | Lung | Yes | 1 | Meropenem | TMP-SMX | - |
| 80 | 53F | Yes | No | DM, CKD | Both (adjacent) | Knee joint, tibia | 2° BJI | Genitourinary | Yes | 3 | Meropenem | TMP-SMX | Pathological fracture, clinically suspected relapse. ^c^ |
| 82 | 52F | Yes | Yes | DM | SA | Knee and ankle joint | 2° BJI | Pulmonary | Yes | 2 | Meropenem | TMP-SMX | - |
| 89 | 53M | Yes | Yes | DM, EtOH | SA | Knee joint | 2° BJI | Lung | Yes | 2 | Meropenem | TMP-SMX | - |
| 107 | 38M | Yes | No | DM, EtOH | SA | Knee joint | 2° BJI | Liver, spleen | Yes | 1 | Ceftazidime | TMP-SMX | - |
| 112 | 55M | Yes | Yes | DM, CLD | Both (adjacent) | Ankle joint, tibia | 2° BJI | Lung | Yes | 1 | Meropenem | TMP-SMX | - |
| 116 | 38M | Yes | Yes | DM | SA | Ankle joint | 2° BJI | Lung | Yes | 3 | Meropenem | TMP-SMX | Readmission for repeat operation for source control |
| 146 | 26M | Yes | Yes | DM, EtOH | SA | Elbow joint | 2° BJI | Lung | Yes | 1 | Ceftazidime | TMP-SMX | Readmission for culture confirmed relapse. |
| 185 | 72M | No | No | DM, CLD | OM | Talus | 2° BJI | Lung | Yes | 0 | Meropenem + ceftazidime | - | Pathological fracture, ADR, death |
| 197 | 57M | No | No | EtOH | Both (adjacent) | Knee joint, femur | 2° BJI | Lung, kidney and prostate | Yes | 1 | Ceftazidime | TMP-SMX | - |
| 205 | 48M | Yes | Yes | DM, CKD | Both (adjacent) | Knee joint, femur | 2° BJI | - | Yes | 7 | Ceftazidime | TMP-SMX | Sinus. Readmission for bone defect requiring Masquelet procedure |
| 224 | 44M | Yes | Yes | DM | OM | Humerus | 2° BJI | - | No | 3 | Ceftazidime | TMP-SMX | - |
| 230 | 56M | No | No | DM | Both (adjacent) | Sacroiliac joint, sacrum | 2° BJI | SSTI | Yes | 0 | Ceftazidime | AMC | ADR |
| 232 | 71F | No | No | DM, CLD | OM | Tibia | 2° BJI | - | Yes | 1 | Meropenem + ceftazidime | TMP-SMX | - |
| 253 | 51N | No | No | Immunosuppression | OM | Humerus | 2° BJI | Lung | No | 2 | Ceftazidime | TMP-SMX | Readmission for repeat operation for source control |
| 282 | 50F | Yes | Yes | DM | OM | Femur | 2° BJI | - | No | 2 | Ceftazidime | TMP-SMX | Readmission for clinically suspected recrudescence. ^d^ |
| 289 | 65M | No | No | DM, EtOH | Both (adjacent) | Knee joint, femur | 2° BJI | Lung, genitourinary, mycotic aneurysm | Yes | 2 | Ceftazidime | TMP-SMX | - |
| 294 | 58F | Yes | No | DM | SA | Shoulder joint | 2° BJI | Genitourinary, spleen, subphrenic collection | No | 1 | Ceftazidime | TMP-SMX | - |
| 304 | 60M | No | No | Nil | OM | Sacroiliac | 2° BJI | Lung | Yes | 0 | Ceftazidime | TMP-SMX | - |
| 313 | 61M | Yes | No | CLD, immunosuppression | OM | Lumbar vertebrae 3 and 4 | 2° BJI | Lung | Yes | 0 | Ceftazidime | Doxycycline | ADR |
| 333 | 46M | No | No | CLD, malignancy, immunosuppression | Both (adjacent) | Knee joint, femur | 2° BJI | Subphrenic collection | Yes | 1 | Ceftazidime | TMP-SMX | Readmission for repeat operation for source control (subphrenic collection) |
| 367 | 77M | No | No | EtOH, immunosuppression | OM | Metatarsal | 2° BJI | Lung, SSTI dorsum foot | Yes | 3 | Ceftazidime | TMP-SMX | - |
| 382 | 47M | Yes | No | DM, EtOH, immunosuppression | Both (non-adjacent) | Ankle joint, elbow joint, metacarpophalangeal joint, talus, metatarsal, tarsometatarsal articulation, cuneiform | 2° BJI | Lung, liver, prostate | Yes | 5 | Ceftazidime | TMP-SMX | - |
| 388 | 45F | Yes | No | DM, EtOH | OM | Tibia | 2° BJI | Lung | Yes | 0 | Ceftazidime | TMP-SMX | - |
| 391 | 69F | Yes | No | DM, CLD, CKD | OM | Femur | 2° BJI | SSTI thigh | Yes | 0 | Meropenem | AMC | Readmission for clinically suspected recrudescence. ^e^ ADR |
| 421 | 56F | No | No | DM | Both (adjacent) | Ankle joint, tibia | 2° BJI | - | Yes | 3 | Ceftazidime + Meropenem | TMP-SMX | Readmission for culture-confirmed recrudescence, non-union, pathological fracture, chronic OM. |

1° BJI; primary bone and joint infection; 2° BJI: secondary bone and joint infection; FNA: First Nations Australian; DM: diabetes mellitus; EtOH: hazardous alcohol use; CLD: Chronic lung disease; CKD: chronic kidney disease; OM: osteomyelitis; SA: septic arthritis; IV: intravenous; PO: oral; TMP-SMX: trimethoprim/sulfamethoxazole; AMC: Amoxycillin and clavulanic acid. SSTI: skin and soft tissue infection; ADR: adverse drug reaction.

^a^ Recurrence of septic arthritis treated at another health service; received > 6/12 of planned 1 year of doxycycline (notes not available)

^b^ Case 69. Clinically suspected recrudescence. Presented initially with culture confirmed melioidosis affecting left ankle, left calcaneus and left femur. He presented 4 months later, while still prescribed TMP-SMX with increasing left hip pain, an elevated c-reactive protein of 125 and an elevated white cell count of 15 x 10^9^/L, with an MRI showing left femur osteomyelitis (the same location as the initial presentation) with abscess formation. The patient declined biopsy and blood cultures were negative. The attending infectious diseases specialist felt recrudescence was likely and the patient was recommenced on IV ceftazidime which he continued for 6 weeks before de-escalation to 6 months of TMP-SMX. He made a complete recovery.

^c^ Case 80. Clinically suspected relapse. Initially presented with left knee septic arthritis with adjacent tibial osteomyelitis. Was prescribed 6 weeks of IV ceftazidime and 6 months of oral TMP-SMX. The patient initially settled but represented 11 months later, 3 months after ceasing antibiotics with recurrent left knee septic arthritis (the same location) and a c-reactive protein of 275 mg/L. Knee washout demonstrated copious pus but was culture negative (the patient was on meropenem at the time of the operation). The attending infectious diseases specialist and orthopaedic surgeon felt this most likely represented relapse of the septic arthritis due to *B. pseudomallei.* The patient was prescribed 2 weeks of IV meropenem and then a further 12 months of TMP-SMX and made a full recovery.

^d^ Case 282. Clinically suspected recrudescence Initially presented with fever, cough, mediastinal lymphadenopathy with *B. pseudomallei* cultured at bronchoscopy. During work-up MRI identified osteomyelitis of right femur with adjacent myositis (non-drainable). The patient received 6 weeks of IV ceftazidime and then oral doxycycline (TMP-SMX was not tolerated due to rash and eosinophilia). An MRI 8 months after presentation showed improved appearance of osteomyelitis but persisting abscesses and a persistently elevated c-reactive protein. A further MRI 3 months later showed persisting changes and as her c-reactive protein remained elevated at 21 mg/L she went on to have surgical debridement of the femur (while still receiving antibiotic therapy). This tissue contained leucocytes but was culture negative (possibly due to the concurrent antibiotic therapy). The attending infectious diseases specialist and the orthopaedic surgeon felt that the operative findings at theatre, the persistent MRI changes and the elevated CRP were all consistent with unresolved *B. pseudomallei* infection.

^e^ Case 391. Clinically suspected recrudescence. A patient on haemodialysis initially presented with cough and pain in her right leg. Blood cultures isolated *B. pseudomallei* and PET/MRI demonstrated osteomyelitis of right femur. She received 6 weeks of IV ceftazidime and was stepped down to oral amoxycillin-clavulanic acid after TMP-SMX caused nausea. While prescribed the oral amoxycillin-clavulanic acid she required an admission to hospital in the remote Torres Straits region with fever, hypotension and an elevated CRP to 118 mg/L. She had blood cultures collected and was commenced on meropenem due to concerns about life-threatening recrudescence. She was transferred down to Cairns Hospital by aeromedical evacuation where MRI of the femur showed persisting changes in the right femur. No other source of infection was identified despite a thorough work up during an 11-day inpatient stay which included 3 blood cultures, urine cultures and a CT chest, abdomen, and pelvis. The attending infectious diseases physician felt that this most likely represented recrudescent melioidosis of her femur. She responded to reinitiation of ceftazidime and then a further 6 months of amoxycillin-clavulanic acid.
