# Supplementary material for: Bone and joint infections due to melioidosis; diagnostic and management strategies to optimise outcomes
Source: PLoS Negl Trop Dis. 2024 Jul 17;18(7):e0012317. doi: 10.1371/journal.pntd.0012317 (PMC11253972; doi:10.1371/journal.pntd.0012317)
Supplement: S2 Table — (DOCX) [file pntd.0012317.s002.docx]

**Supplementary table 2**: Characteristics of the individuals with bone and joint infections due to *B. pseudomallei*, stratified by clinical phenotype.

| Risk factor | All  (n = 39) | Only osteomyelitis (n = 14) | Only septic arthritis (n = 8) | Both osteomyelitis and septic arthritis (n = 17) |
| --- | --- | --- | --- | --- |
| Age | 52 (42-57) | 56 (45-70) | 46 (38-57) | 48 (38-56) |
| Male | 28 (72%) | 10 (71%) | 4 (50%) | 14 (82%) |
| First Nations Australian | 26 (67%) | 8 (57%) | 8 (100%) | 10 (59%) |
| Remote residence | 20 (51%) | 5 (36%) | 6 (75%) | 9 (53%) |
| Wet season presentation | 25 (64%) | 9 (64%) | 6 (75%) | 10 (59%) |
| Acute presentation | 34 (87%) | 11 (79%) | 6 (75%) | 17 (100%) |
| Chronic presentation | 5 (13%) | 3 (21%) | 2 (25%) | 0 |
| Bacteraemic | 31 (79%) | 10 (71%) | 6 (75%) | 15 (88%) |
| Diabetes mellitus | 31 (79%) | 9 (64%) | 8 (100%) | 14 (82%) |
| Hazardous alcohol consumption | 15 (38%) | 4 (29%) | 3 (38%) | 8 (47%) |
| Chronic lung disease | 7 (18%) | 4 (29%) | 0 | 3 (18%) |
| Immunosuppression | 6 (18%) | 3 (21%) | 0 | 3 (18%) |
| Chronic kidney disease | 3 (8%) | 1 (7%) | 0 | 2 (12%) |
| Malignancy | 1 (3%) | 0 | 0 | 1 (7%) |
| Total number of predisposing risk factors | 2 (1-2) | 2 (1-2) | 1 (1-2) | 2 (1-3) |
| No risk factors for melioidosis | 2 (5%) | 2 (14%) | 0 | 0 |
| Isolated BJI | 5 (13%) | 1 (7%) | 0 | 4 (24%) |
| ICU admission | 11 (28%) | 2 (14%) | 5 (63%) | 4 (24%) |
| Death in hospital due to melioidosis | 1 (3%) | 1 (7%) | 0 | 0 |
| Data presented as n (%) or median (interquartile range), as appropriate.  BJI: Bone and joint infection. ICU: Intensive Care Unit. | | | | |
