# Supplementary material for: Bone and joint infections due to melioidosis; diagnostic and management strategies to optimise outcomes
Source: PLoS Negl Trop Dis. 2024 Jul 17;18(7):e0012317. doi: 10.1371/journal.pntd.0012317 (PMC11253972; doi:10.1371/journal.pntd.0012317)
Supplement: Attachment — Submitted filename: PNTD-D-24-00546 response to reviewers.docx [file pntd.0012317.s003.docx]

**Response to reviewers**

We thank the Editorial staff and the reviewers for the time that they have taken to review our manuscript and the very helpful comments that they have made to improve the work. Please find below our point-by-point responses (in black) to their comments (in blue). We note that there was significant disparity in the reviewers’ comments: reviewers 2 and 3 were very positive and had few suggestions for the paper’s enhancement. While reviewer 1 and 4 were less positive; we have our best in the revised manuscript to address their concerns, although reviewer 4 offered few specific criticisms.

Reviewer's Responses to Questions

Key Review Criteria Required for Acceptance?
As you describe the new analyses required for acceptance, please consider the following:

Methods
-Are the objectives of the study clearly articulated with a clear testable hypothesis stated?
-Is the study design appropriate to address the stated objectives?
-Is the population clearly described and appropriate for the hypothesis being tested?
-Is the sample size sufficient to ensure adequate power to address the hypothesis being tested?
-Were correct statistical analysis used to support conclusions?
-Are there concerns about ethical or regulatory requirements being met?

Reviewer #1: Comments as below

Reviewer #2: (No Response)

Reviewer #3: Study population well defined.  Retrospective study, goal to assess outcomes
Study does not lend itself to a hypothesis
No concern about ethics:  Data were retrospective and de-identified, the Committee waived the requirement for informed consent.

Reviewer #4: (No Response)

**Response:** We are pleased that reviewer 3 was happy with the methods that we employed in the paper.

--------------------

Results
-Does the analysis presented match the analysis plan?
-Are the results clearly and completely presented?
-Are the figures (Tables, Images) of sufficient quality for clarity?

Reviewer #1: Comments as below

Reviewer #2: (No Response)

Reviewer #3: Images and figures well done

Reviewer #4: (No Response)

**Response:** We are happy that reviewer 3 was happy with the results section and figures in the paper.

--------------------

Conclusions
-Are the conclusions supported by the data presented?
-Are the limitations of analysis clearly described?
-Do the authors discuss how these data can be helpful to advance our understanding of the topic under study?
-Is public health relevance addressed?

Reviewer #1: Comments as below

Reviewer #2: (No Response)

Reviewer #3: Limitations clearly outline - retrospective study, small number of patients affected and no standardized documentation of operative strategy

Reviewer #4: (No Response)

**Response:** We are pleased that reviewer 3 was happy that we have outlined the limitations of the paper clearly.

--------------------

Editorial and Data Presentation Modifications?
Use  this section for editorial suggestions as well as relatively minor modifications of existing data that would enhance clarity. If the only modifications needed are minor and/or editorial, you may wish to recommend “Minor Revision” or “Accept”.

Reviewer #1: Comments as below

Reviewer #2: (No Response)

Reviewer #3: Accept

Reviewer #4: (No Response)

**Response:** We are happy that reviewer 3 feels that the paper should be accepted.

--------------------

Summary and General Comments
Use  this section to provide overall comments, discuss strengths/weaknesses of the study, novelty, significance, general execution and scholarship. You may also include additional comments for the author, including concerns about dual publication, research ethics, or publication ethics. If requesting major revision, please articulate the new experiments that are needed.

Reviewer #1: This is a well written retrospective review of Bone and joint infections (BJI) caused by Burkholderia pseudomallei , in Far North Queensland. It covers about 25 years and a comparative analysis is provided against those cases of infection with this organism where there was no evidence of a BJI.

The main concern that I have with this study is that it compares a very uncommon cause of a BJI (39 cases over 25 years – 1-2 cases a year) with other patients with melioidosis. It really should have compared this cause of a BJI, with the far commoner cause of a BJI – namely Staph aureus. This is a significant limitation of the study. As it is, it offers little that is new. What is needed is how infection of bone or joint with Burkholderia pseudomallei,  differs both epidemiologically and clinically from that caused by Staphylococcus aureus (at the very least).

**Response:** We thank reviewer 1 for the time that they have taken to review our paper and their constructive suggestions for its enhancement.

The reviewer is correct to note that we present a relatively uncommon cause of BJI, but the manuscript is the fourth largest series of *B. pseudomallei* BJI ever presented in the literature and the second largest from high-income countries (where clinicians have access to advanced imaging including MRI and PET-CT and well-resourced surgical support in a universal health system). The only larger study from a high-income country was the paper, published almost 10 years ago by Shetty and colleagues who described 50 cases managed at the Royal Darwin Hospital dating back to 1989 when advanced imaging was not available at that facility. Indeed, there is no discussion of any imaging findings in that paper. We also report a 90-day mortality of 0% (the only patient to die in the series was a highly comorbid patient who died from respiratory failure 138 days after his presentation). This is only the third series – and the largest – to report a 90-day case-fatality rate of 0%.

This is why we have submitted the paper to the open access journal PLoS Neglected Tropical Diseases. The paper allows clinicians from less well-resourced health systems who encounter melioidosis to gain greater insights into the pathophysiology of *B. pseudomallei* BJI and to see the clinical and diagnostic strategies – that they may have less experience with – that are associated with positive outcomes. PLoS NTD is devoted to the study of neglected tropical diseases, that are endemic to tropical regions and melioidosis is one of the priority bacterial infections that is within the scope of journal. If we were going to examine BJI due to *Staphylococcus aureus,* *Pseudomonas aeruginosa* and other pathogens, this would not be within the scope of the journal (we would instead submit the paper to a dedicated journal of infectious diseases or orthopaedics). This would also be a much, much larger study requiring a separate ethics approval.

The sole focus on *B. pseudomallei* BJI allows us to examine the epidemiological, clinical, microbiological and radiological details of BJI due this pathogen and to correlate them, in some depth, with the subsequent clinical course. As a result, we have been able to examine several aspects of BJI due to *B. pseudomallei* that have not been explored in depth in prior papers to address the subject (table 1 below).

For instance, we were able to confirm not only that diabetes was common in patients with BJI due to *B. pseudomallei*, by examining patients with melioidosis with ‒ and without ‒ BJI we were able to demonstrate an independent association between BJI and diabetes mellitus (rather than any other demographic or clinical epidemiological associations) in one of the world’s largest databases of melioidosis cases. This is first paper ever to do this and suggests an important role of diabetes in the pathophysiology of not only melioidosis, but BJI due to melioidosis.

The paper is also the first to compare, in detail, the clinical course and management of septic arthritis and osteomyelitis (previous papers have tended to regard septic arthritis and osteomyelitis as a single entity), the first to present PET images and only the second to present MRI images. The paper also highlights that BJI is usually part of multi-organ involvement with almost 75% of the patients in our cohort presenting with other manifestations of melioidosis and highlighting that BJI may not be apparent on initial presentation. This is an important distinction as all but one previous series have focussed on the BJI in the patient, when in most cases it is simply part of multi-organ involvement (and which is likely to explain the signification variation in the CFR in different series).

It would not be possible to present this detailed analysis of BJI due to *B. pseudomallei* if we were also having to present the comparative epidemiological, clinical, microbiological, and radiological details of BJI due to other pathogens.

**Table 1.** The focus on BJI due to *B. pseudomallei* enabled us to concentrate on aspects of the infection that had not been examined in previous papers to address the subject.

| Authors | Year | Country | n | MRI images | PET images | Is DM an independent risk factor for BJI in patients with melioidosis | Analysis of BJI as a primary or secondary feature of presentation | Detailed comparison of SA and OM | Detailed review of relapse, recrudescence complications |
| --- | --- | --- | --- | --- | --- | --- | --- | --- | --- |
| Kosuwon *et al.* | 2003 | Thailand | 25 | No | No | No ^a^ | No | No | **Yes** |
| Pande *et al.* | 2011 | Brunei | 8 | No | No | No | No | Limited | **Yes** |
| Shetty *et al.* | 2015 | Australia | 50 | No | No | No | **Yes** | Limited | **Yes** |
| Teparrukkul *et al*. | 2017 | Thailand | 74 | No | No | No ^a^ | No | No | **Yes ^b^** |
| Zueter et al. | 2017 | Malaysia | 19 ^c^ | No | No | No | No | No | No |
| Gupta *et al.* | 2021 | India | 11 | **Yes** | No | No | No | No | No |
| Wu *et al*. | 2021 | China | 44 | No | No | No | No | Limited | Limited |
| **Current study** | **2024** | **Australia** | **39** | **Yes** | **Yes** | **Yes** | **Yes** | **Yes** | **Yes** |

MRI: Magnetic resonance imaging; PET: positron emission tomography; DM: diabetes mellitus; BJI: Bone and joint infection.

^a^ Did highlight that patients in Northern Thailand with BJI and diabetes were more likely to have melioidosis than a BJI due to another pathogen but did not determine the relative likelihood of BJI involvement in patients with melioidosis.

^b^ Incomplete follow up; data of long-term outcomes were available only 48% of patients

^c^ Also included soft tissue infection due to melioidosis; only 9/19 (47%) had bone and joint involvement

However, we do highlight the relatively high relapse and recrudescence rates and an absence of BJI associated with prosthetic material and the reviewer is absolutely correct to suggest that we could have developed this more. In the revised manuscript we have expanded the discussion significantly to address these concerns (lines 341-348).

Furthermore, while we were not able to directly compare the clinical course of patients with BJI due to other pathogens at our hospital over the same time period (for the reasons outlined above) we have compared our findings with large contemporary series of BJI from other well-resourced settings (lines 444-454). Here we demonstrate that we had a higher readmission rate in our series than in a large French series (reference 19, where almost a third of patients had more complex BJI which involved prosthetic material). We also demonstrate that we had a similar treatment failure rate to a large UK series (reference 48, where again almost a quarter of the patients retained their prosthetic material) despite the fact that our patients received a median of > 7 months of antibiotic therapy compared to a median of < 80 days in the UK series. These comparisons support our assertion that BJI due to *B. pseudomallei* may have a more complicated course than BJI due to other pathogens.

In the revised submission, we have also highlighted the lack of direct comparison with other pathogens as a limitation of the study (lines 477-478) and have provided suggestions for the focus of future studies to examine the subject (lines 476-484).

Other points are

Line 156:  This is too vague, either the organism was re-isolated and a decision made to restart treatment (relapse) or, there was failure to clinically improve during therapy which was then prolonged. This should not be called recrudescence unless the organism was reisolated during treatment.

**Response:** We thank the reviewer for raising this issue. Recrudescence and relapse are commonly used terms in the melioidosis literature (<https://pubmed.ncbi.nlm.nih.gov/25811783/>). In our study, recrudescence was defined as recurrence during the period that the patient was prescribed antibiotic therapy; relapse was defined as recurrence after completion of their prescribed antibiotic therapy (lines 154-156).

We agree that microbiological confirmation of *B. pseudomallei* infection is required for a diagnosis of melioidosis and all 477 cases in our series (including the 39 patients with BJI) had the diagnosis of melioidosis confirmed with culture.

There were 5/39 patients with BJI who had recrudescence (2 culture confirmed, 3 suspected clinically) and 3 additional patients who had relapse (2 culture confirmed, 1 suspected clinically).

In the 4 cases where recrudescence and relapse were clinically suspected but not microbiologically confirmed, there was consensus from the attending orthopaedic consultant and infectious diseases physician, after reviewing biochemical, imaging and in some cases operative findings, that recrudescence or relapse was likely. In the revised submission we have added detailed descriptions to supplementary table 1 to help the reader understand why recrudescence/relapse was felt to be likely.

We have added them here for the reviewer and Editor to review:

Case 69. Clinically suspected recrudescence. Presented initially with culture confirmed melioidosis affecting left ankle, left calcaneus and left femur. He presented 4 months later, while still prescribed TMP-SMX with increasing left hip pain, an elevated c-reactive protein of 125 and an elevated white cell count of 15 x 10^9^/L, with an MRI showing left femur osteomyelitis (the same location as the initial presentation) with abscess formation. The patient declined biopsy and blood cultures were negative. The attending infectious diseases specialist felt recrudescence was likely and the patient was recommenced on IV ceftazidime which he continued for 6 weeks before de-escalation to 6 months of TMP-SMX. He made a complete recovery.

Case 80. Clinically suspected relapse. Initially presented with left knee septic arthritis with adjacent tibial osteomyelitis. Was prescribed 6 weeks of IV ceftazidime and 6 months of oral TMP-SMX. The patient initially settled but represented 11 months later, 3 months after ceasing antibiotics with recurrent left knee septic arthritis (the same location) and a c-reactive protein of 275 mg/L. Knee washout demonstrated copious pus but was culture negative (the patient was on meropenem at the time of the operation). The attending infectious diseases specialist and orthopaedic surgeon felt this most likely represented relapse of the septic arthritis due to *B. pseudomallei.* The patient was prescribed 2 weeks of IV meropenem and then a further 12 months of TMP-SMX and made a full recovery.

Case 282. Clinically suspected recrudescence Initially presented with fever, cough, mediastinal lymphadenopathy with *B. pseudomallei* cultured at bronchoscopy. During work-up, MRI identified osteomyelitis of right femur with adjacent myositis (non-drainable). The patient received 6 weeks of IV ceftazidime and then oral doxycycline (TMP-SMX was not tolerated due to rash and eosinophilia). An MRI 8 months after presentation showed improved appearance of osteomyelitis but persisting abscesses and a persistently elevated c-reactive protein. A further MRI 3 months later showed persisting changes and as her c-reactive protein remained elevated at 21 mg/L she went on to have surgical debridement of the femur (while still receiving antibiotic therapy). This tissue contained leucocytes but was culture negative (possibly due to the concurrent antibiotic therapy). The attending infectious diseases specialist and the orthopaedic surgeon felt that the operative findings at theatre, the persistent MRI changes and the elevated CRP were all consistent with unresolved *B. pseudomallei* infection.

Case 391. Clinically suspected recrudescence. A patient on haemodialysis initially presented with cough and pain in her right leg. Blood cultures isolated *B. pseudomallei* and PET/MRI demonstrated osteomyelitis of right femur. She received 6 weeks of IV ceftazidime and was stepped down to oral amoxycillin-clavulanic acid after TMP-SMX caused nausea. While prescribed the oral amoxycillin-clavulanic acid she required an admission to hospital in the remote Torres Straits region with fever, hypotension and an elevated CRP to 118 mg/L. She had blood cultures collected and was commenced on meropenem due to concerns about life-threatening recrudescence. She was transferred down to Cairns Hospital by aeromedical evacuation where MRI of the femur showed persisting changes in the right femur. No other source of infection was identified despite a thorough work up during an 11-day inpatient stay which included 3 blood cultures, urine cultures and a CT chest, abdomen, and pelvis. The attending infectious diseases physician felt that this most likely represented recrudescent melioidosis of her femur. She responded to reinitiation of ceftazidime and then a further 6 months of amoxycillin-clavulanic acid.

Line 168   The results section could be reduced significantly as data is repeated and could be referred to in the tables.

**Response:** We thank the reviewer for raising this point. As highlighted above, we were keen to present the epidemiological, clinical, microbiological, and radiological characteristics in some detail to help the reader have greater insight into this rare manifestation of melioidosis. However, there is usually only a single paragraph on each of these. We were also at pains to limit repeating our data: for instance, only the multivariate analysis of the associations between patient characteristics and BJI due to *B. pseudomallei* are presented in the text, while the univariate analysis is presented in table 1. Most of the detailed data (which we believe is important to include) has been relegated to supplementary material (supplementary tables 1 and 2). We would be happy to relegate more of the data from the results section to supplementary files if the Editor feels that this improves the paper.

Line 263  Once again this is of little value if it depends on clinical opinion. If there is something unusual about either relapse/recrudescence of a BJI with melioidosis as opposed to say Staph aureus, then it should be mentioned and referenced. If not then this statement should be restricted to cases where the organism was reisolated and proven to be identical.

**Response:** It is absolutely reasonable for the reviewer to seek clarification here. We have now described, in detail, the 4 cases where the recrudescence or relapse was only clinically suspected, not microbiologically confirmed. In all 4 cases there was clinical and radiological evidence of persisting infection in the site of the original infection and 2/4 went to theatre where operative findings were also consistent with recrudescence or relapse.

There were also extenuating circumstances that meant a positive culture was less likely: case 69 declined a biopsy; case 80 had copious pus in her keen joint at operation but was on meropenem at the time of the procedure, reducing the likelihood of culturing the organism; case 282 had operative findings consistent with osteomyelitis but was on meropenem at the time of the procedure, reducing the likelihood of culturing the organism; case 391 had an 11-day inpatient admission where no other source of infection was identified despite extensive radiological and microbiological evaluation. She had presented febrile and hypotensive to a peripheral hospital 800km from the nearest tertiary centre; given the recent episode of melioidosis it was reasonable to start her empirically on meropenem although reduced the likelihood of a positive culture and she did not have debridement of the osteomyelitis (she was a patient on haemodialysis with significant comorbidity and so an initial non-operative approach was pursued; fortunately, this was successful in achieving cure).

Line 323  How many of all Melioidosis cases had a prosthetic joint that was NOT infected? If unknown then this statement cannot be made

**Response:** We respectfully disagree with the reviewer on this point. Joint replacement is a common procedure in Australia: the prevalence of joint replacement in Australians is 22.5%, and 13.3% in those aged >85 years and 65-84 years, respectively and joint infection is the most common complication and, sadly, a frequent issue in our hospital.

In 2022, 126 000 knee and hip replacements were performed in Australia, adding to the pool of about 1 million Australians currently living with one or more joint replacements in situ. Unfortunately, around 4000 Australians are diagnosed with a prosthetic joint infection annually. The absence of a single BJI involving prosthetic material over the 25 years of this study in a population of over 290,000 where joint replacement and orthopaedic implants are everyday events is therefore a striking finding.

In a very large series from another high-income setting (France) 11448/36091 (31.7%) BJIs involved prosthetic material; the fact that 0/39 patients in our series had prosthetic material is noteworthy (and, although the cohorts are not directly comparable, statistically significant to a p value of <0.0001).

Line 386 and 396  Is this unique to B.pseudomallei?

**Response:** We thank the reviewer for this comment.

Line 386: “Our cohort shows that when BJI is present it is also often multifocal and that if septic arthritis is present, the adjacent bone is also frequently involved”

**Response**: This may not be unique to *B. pseudomallei* however the finding that 4/39 (10%) had multifocal involvement of non-adjacent sites is, we believe, worth highlighting, to ensure that clinicians actively seek BJI in other sites. Unrecognised or persisting osteomyelitis has been linked to relapse and recrudescence in other series (<https://pubmed.ncbi.nlm.nih.gov/32986699/>).

Although septic arthritis with adjacent osteomyelitis is seen more frequently in children, it is a relatively uncommon occurrence in adults; it was a finding in no fewer than 17/39 (44%) cases in this series which we feel is highly notable.

Line 396: “This is particularly true for melioidosis, as persisting osteomyelitis has been attributed to an increased risk of melioidosis recrudescence.”

**Response**: Recrudescence and relapse may be life-threatening in patients with melioidosis. In the landmark Darwin Prospective Melioidosis Series (DPMS), after non-adherence to eradication therapy was excluded, unrecognised or inadequately controlled BJI was the commonest reason for disease recrudescence and relapse (<https://pubmed.ncbi.nlm.nih.gov/32986699/>). While clearly, disease recrudescence and relapse can also occur with other pathogens, relapse is seen much more commonly in melioidosis than with other organisms (almost 10% of cases of melioidosis experienced relapse in one Thai series <https://pubmed.ncbi.nlm.nih.gov/16983608/>). As a result, even after aggressive source control, extended courses of intravenous (minimum of 6 weeks) and then oral antibiotics (minimum of 6 months) are recommended for Australians with BJI due to *B. pseudomallei*. In contrast for acute osteomyelitis in adults due to other pathogens, national guidelines recommend as little as 6 weeks of total (IV and oral) antibiotic therapy if patients have a good clinical response.

Reviewer #2: This is an extremely well written case series involving 39 patients with melioidosis and bone and/or joint involvement.  Excellent outcomes were achieved, which would not necessarily have been the case in many less well-developed settings, and I think that both this and the vital importance of early surgical referral could have been emphasised more strongly.

**Response:** We thank the reviewer for the time that that he/she has taken to review our work and the constructive suggestions for its improvement. We agree that we could have expanded our discussion about the importance of early surgical referral. In the revised submission we have specifically highlighted the importance of early surgical review in the abstract, the author’s summary and the conclusions (lines 54-55, 75 and 546).

Otherwise, I have only a couple of suggestions.

1.      Was genotyping done in the 3 patients with culture-positive recurrence, to confirm labelling the 2 post-treatment recurrences as relapse as opposed to reinfection (although the latter is extremely unlikely). This should be made clear.

**Response:** The reviewer raises an important point. Unfortunately, genotyping (whole-genome sequencing) was only available after 2022 and so we could not confirm recurrence (rather than re-infection) in the 4 patients with culture-confirmed relapse. However, in all 4 the infection recurred at the site of the initial infection suggesting that recurrence was the more likely explanation. We have added some text to highlight this (lines 160-161, 273-276).

2.      Figure 1 should be labelled ‘ulna’ rather than ‘ulnar’.

**Response:** We thank the reviewer for noting this typographical error, we have amended the figure accordingly.

Reviewer #3: This is a very well written manuscript.  The authors link risk for meliodosis to presence of diabetes, but given median hemoglobin a1c of 10.3 I perhaps the comment should be about poorly controlled diabetes.  May also comment on high rates of tobacco use and more clearly define "hazardous" alcohol use.  Do the authors want to comment on why plain radiographs performed so poorly and why there is a strong male predominance?

**Response:** We thank the reviewer for the time that that he/she has taken to review our work and the constructive suggestions for its improvement.

We agree with the reviewer’s suggestion that we should have highlighted poorly controlled diabetes as a risk for the disease. Of 31 individuals with diabetes mellitus, only 2 had a glycosylated haemoglobin below the target of 7% and 80% had an HbA1c > 9%. Poorly controlled diabetes is more likely to result in end organ micro- and macrovascular complications which we hypothesise may explain the higher incidence of BJI in the lower limb. We have amended the text accordingly (lines 181-193, 325-326).

We agree that there were relatively high rates of tobacco use and hazardous alcohol use in the cohort. We have previously demonstrated that melioidosis is a disease of disadvantaged populations in our region (https://pubmed.ncbi.nlm.nih.gov/34153059/ ) and in our region hazardous alcohol use and tobacco use is more common in these populations (<https://www.choreport.health.qld.gov.au/>).

Alcohol use is, of course, one of the most common predisposing conditions for melioidosis which at least partly explains the high prevalence of hazardous alcohol use in the cohort. We have explained how we defined hazardous alcohol use in previous analyses of this cohort (reference 13, lines 134-136). Fundamentally, this is based on documentation of hazardous alcohol use in the medical record in the prior 12 months which is based on the current Australian National Health and Medical Research Council (NHMRC) guidelines (<https://www.nhmrc.gov.au/health-advice/alcohol>) that suggest that >10 standard drinks a week represents hazardous consumption.

We have presented this data in previous publications and described our definitions in references in this article (references 3 and 13) and so we have not presented them again here, however, we would be happy to add this to the methods if the Editor feels that it enhances the paper.

The poor sensitivity of plain x-ray for osteomyelitis has at least two explanations. The first is that plain x-ray is an intrinsically less sensitive modality for subtle bony changes than alternatives such as MRI (although of course plain x-ray is widely available, easier to access, and more easily interpreted by non-radiologists). The second is that the changes of acute osteomyelitis may not be evident in adults for 10-14 days. As all 17 patients with both osteomyelitis and septic arthritis had an acute presentation and 11/14 (79%) of patients with osteomyelitis alone had an acute presentation, radiological changes may not have been apparent on initial x-ray (which are almost always the first imaging modality). CT and MRI imaging was almost always performed later in the patients’ hospitalisation, meaning that more time had elapsed for radiological changes to become apparent. As isolated septic arthritis has no bony involvement, plain x-ray will be less sensitive for this condition. We have expanded the discussion to highlight some of these points (lines 380-382). We have also added a new figure (Figure 3) to highlight that the plain x-ray can be normal despite significant symptoms and an underlying osteomyelitis that is visible on more sophisticated imaging.

A male predominance is present in almost all series of melioidosis. This is likely to be linked to a higher incidence of some predisposing comorbidities (for instance hazardous alcohol use) and also as a result of greater potential exposure to the environmental pathogen (that resides in soil and surface water) through occupational or recreational activities.

Reviewer #4: The manuscript briefs the clinical presentation and outcomes of BJI in Melioidosis infected patients . Although well written the paper does not have any novel findings to report , and the number of cases are too less to conclude anything…

**Response:** We thank A/Professor Shaw for the time that that he has taken to review our work although we are disappointed that he feels that there were no novel findings, and the case series is too small.

We would respectively suggest that we have spent some time examining novel elements of BJI due to *B. pseudomallei* as we describe above in our response to reviewer 1.

Specifically, this is the first paper to examine the *independent* contribution of diabetes mellitus to the presence of BJI in patients (identifying a significant association). The study also is the first, to our knowledge, to compare the epidemiological and clinical characteristics of individuals with septic arthritis due to *B. pseudomallei,* osteomyelitis due to *B. pseudomallei* and individuals with both septic arthritis and osteomyelitis (supplementary table 2). It is (now, in this revised submission) also the first the present PET images and discuss their utility in expediting the diagnosis.

It is true that the case series is relatively small when compared to series of other manifestations of *B. pseudomallei* infection, but it is the 4^th^ largest study of BJI complicating melioidosis in the literature and it is, we would argue, the most detailed to examine the subject. It is also the first from a high-income setting for almost 10 years and incorporates up to date knowledge about the use of medical imaging (include MRI and PET) in this patient cohort.

Of course, BJI is a relatively uncommon manifestation of melioidosis. In the DPMS, only 10% of patients with melioidosis developed a BJI. It is, perhaps, for this reason why a detailed examination of the epidemiological, clinical, microbiological and radiological details of BJI due to *B. pseudomallei* is welcome. We hope that clinicians working in endemic areas ‒ particularly those in resource-limited settings ‒ who may still encounter BJI due to *B. pseudomallei* infrequently, will find our detailed descriptions helpful in the management of their patients.
